# The use of 3D additive manufacturing technology in autogenous dental transplantation

**DOI:** 10.1186/s41205-020-00070-9

**Published:** 2020-07-24

**Authors:** Pau Cahuana-Bartra, Abel Cahuana-Cárdenas, Lluís Brunet-Llobet, Marta Ayats-Soler, Jaume Miranda-Rius, Alejandro Rivera-Baró

**Affiliations:** 1 Department of Pediatric Dentistry, Hospital Sant Joan de Déu, University of Barcelona, Passeig Sant Joan de Déu 2, Esplugues de Llobregat, 08950 Barcelona, Spain; 2 Department of Odontostomatology, Faculty of Medicine and Health Sciences, University of Barcelona, Barcelona, Spain; 3 Hospital Dentistry, Clinical Orthodontics and Periodontal Medicine Research Group (HDCORPEMrg), Institut de Recerca Sant Joan de Déu (IRSJD), Barcelona, Spain; 4 Unit of Three-Dimentional Printing (3DP), Innovation Department, Fundació Sant Joan de Déu (FSJD), Barcelona, Spain

**Keywords:** Tooth autotransplantation, Additive manufacturing, Dental ectopy, Polyjet 3D, Dental replica

## Abstract

**Background:**

In medicine and dentistry, 3D technology allows the virtual planning and printing of surgical replicas of anatomical structures that can facilitate certain transplant procedures. In dentistry, 3D technology is useful in autogenous tooth transplantation.

**Case presentation:**

We present a clinical case of an ectopic mandibular second premolar, describing the preoperative planning with dental replicas and the autotransplantation surgery. 3D prints of the surgical replica of the tooth to be transplanted was made using an Objet30 Prime® Printer, PolyJet. Clinical controls performed at 3, 6 and 12 months indicated the satisfactory evolution of the transplanted tooth.

**Conclusion:**

3D additive manufacturing technology allows the preparation of a new recipient socket with the aid of a surgical replica of the tooth to be transplanted, thus minimizing handling and extraoral time.

## Background

Ectopic tooth eruption is an alteration of the eruptive process that most frequently affects the third molars and the upper canines [1–3].

The mandibular second premolar (2 PM) is one of the last permanent teeth to erupt. Its eruption may be delayed, especially when associated with the agenesis of a permanent tooth [4]. In this situation, the 2 PM lacks sufficient space in the arch and so there is an increased risk of malocclusions such as crowding, ectopy or impaction. The etiology of ectopic 2 PM remains unknown and its frequency is estimated at around 0.2–0.3%. Dental abnormalities such as agenesis, microdontia, developmental delay, canine palatal ectopia, and ankylosis often coincide with the distal angulation of 2 PM [5–7]. In 2009, all these combinations of alterations were grouped together under the umbrella term “dental anomaly patterns” and they are found in almost 25% of orthodontic patients [8, 9].

The treatment of ectopic tooth eruption aims to relocate the tooth in its anatomical position. It involves surgical exposure and subsequent orthodontic traction. In severe cases with poor prognosis, autogenous tooth transplant is considered as a therapeutic alternative to surgical removal of the ectopic tooth.

In autogenous tooth transplantation, a recipient socket must be created for the insertion of the donor tooth. The tooth to be transplanted is extremely vulnerable, especially during the examination of its fit inside the new alveolar bed, when injury to the periodontal ligament is inevitable. Furthermore, whenever possible, the extraoral handling time of the donor tooth should be kept to a minimum, since it negatively affects the tooth’s viability [10].

In order to minimize damage to the tooth to be transplanted, helical CT/cone beam-computed tomography is used in combination with computer-aided rapid prototyping [11]. The process named 3D additive manufacturing technology consists of building a three-dimensional object directly using a 3D model in any type of file (e.g., STL, 3MF, STP), usually by the successive addition of material layer upon layer [12]. This technology allows the printing of dental replicas for use as guides during surgery, thus minimizing extraoral time and limiting probable damage to the periodontal tissue of the donor tooth [11]. All of this favors the creation of a new recipient socket, avoiding risks to the donor tooth and, more generally, helping to standardize the procedure [13, 14].

Various 3D printing systems are available, among them stereolithography apparatus (SLA), fused deposition modeling (FDM), selective laser melting (SLM), direct metal laser sintering (DMLS) and material jetting (Polyjet) [13]. The main advantages and technological characteristics of each system are shown in Table 1 [15].

**Table 1:** Classification of additive manufacturing technology

Additive Manufacturing Technology	Materials	Characteristics	Advantages
**SLA** (Stereolithography Apparatus)	POLYMERS	An object is created by selectively curing a polymer resin layer-by-layer using an ultraviolet (UV) laser beam. The materials used in SLA are photosensitive thermoset polymers that come in a liquid form.	* Polymers that come in a liquid form very high dimensional accuracy and with intricate details * SLA parts have a very smooth surface finish, making them ideal for visual prototypes * Speciality SLA materials are available, such as clear, flexible and castable resins
**FDM** (Fused Deposition Modeling)	POLYMERS CERAMICS	An object is built by selectively depositing melted material in a pre-determined path layer-by-layer. The materials used are thermoplastic polymers and come in a filament form.	* The most cost-effective way of producing customized thermoplastic parts and prototypes. * Wide range of materials available; from commodity thermoplastics to engineering materials and high-performance thermoplastics.
**SLM** (Selective laser Melting) & **DMLS** (Direct Metal Laser Sintering)	METALS	They belong to a powder bed fusion technology that uses a laser beam to fuse metal powder layer by layer. SLM produces parts from a single metal DMLS produces parts from metal alloys.	* Superalloy with excellent wear and corrosion resistance. Excellent mechanical properties at high temperatures. * Its applications are: Aerospace and medical (implants) production parts.
**POLYJET** (Material jetting)	POLYMERS	Operates in a similar fashion to 2D printers in material jetting, a print head dispenses droplets of a photosensitive material that solidifies under ultraviolet light, building a part layer-by-layer. The materials (acrylics) that come in a liquid form used in material jetting are thermoset photopolymers	* Can produce smooth parts with surfaces comparable to injection molding and very high dimensional accuracy. * Parts created with material jetting have homogeneous mechanical and thermal properties. * The multi-material capabilities of material jetting enables the creation of accurate visual and realistic prototypes

Here we describe a case of autogenous tooth transplant using replicas of the donor tooth, 3D printed with Polyjet technology, for use as a surgical guide in the creation of the recipient socket.

## Case presentation

**Fig. 1:**
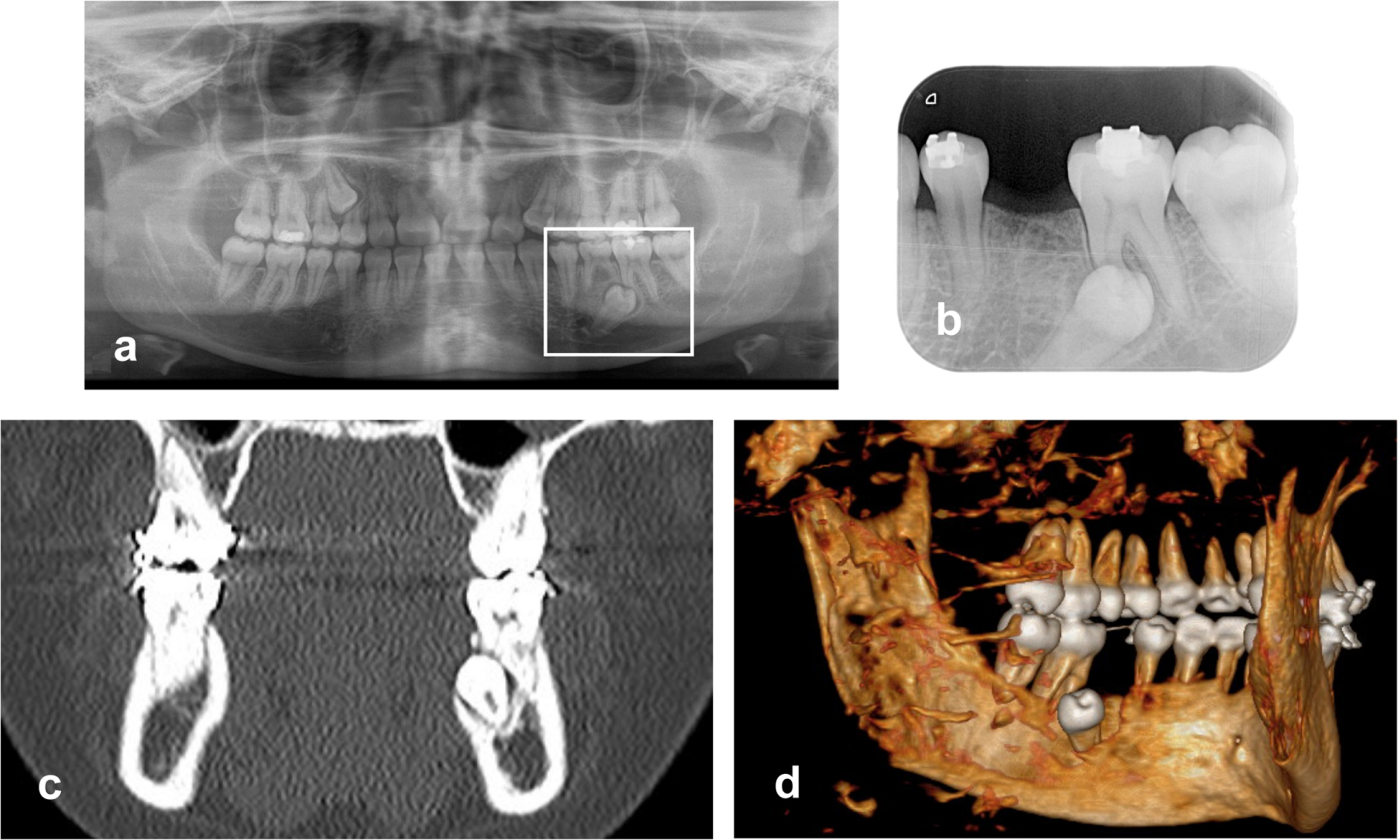
Pre-intervention. **a** Orthopantomography; **b** Periapical x-ray 4 months after extraction of tooth 75; **c** and **d** CT showing the position of tooth 35 (lingual view)

A 16-year-old patient with no medical history of interest attended the Orthodontic Service of the Hospital St Joan de Déu in due to transposition of teeth 13 and 14 (FDI notation) and persistence of tooth 75. Panoramic radiography showed tooth 35 to be impacted, in lingual direction, and oriented distally, close to the mesial root of tooth 36. Orthodontic treatment of the discrepancy consisted of the extraction of tooth 14 to allow eruption of canine 13, and extraction of tooth 75 to allow spontaneous eruption of tooth 35. However, 4 months after the extraction, tooth 35 remained in its ectopic position (Fig. 1).

An alveolotomy was then performed in tooth 35 to facilitate its eruptive process and orthodontic traction. Four months later, an X-ray revealed a dehiscence of the lingual cortical of tooth 35, but no change from its original position.

Before surgical excision, it was decided to plan the autotransplant of tooth 35 to a new recipient socket with the help of a 3D printed dental replica. This replica was to be used as a surgical guide to prepare the alveolar bed, thus minimizing the handling of the donor tooth during its relocation.

**Fig. 2:**
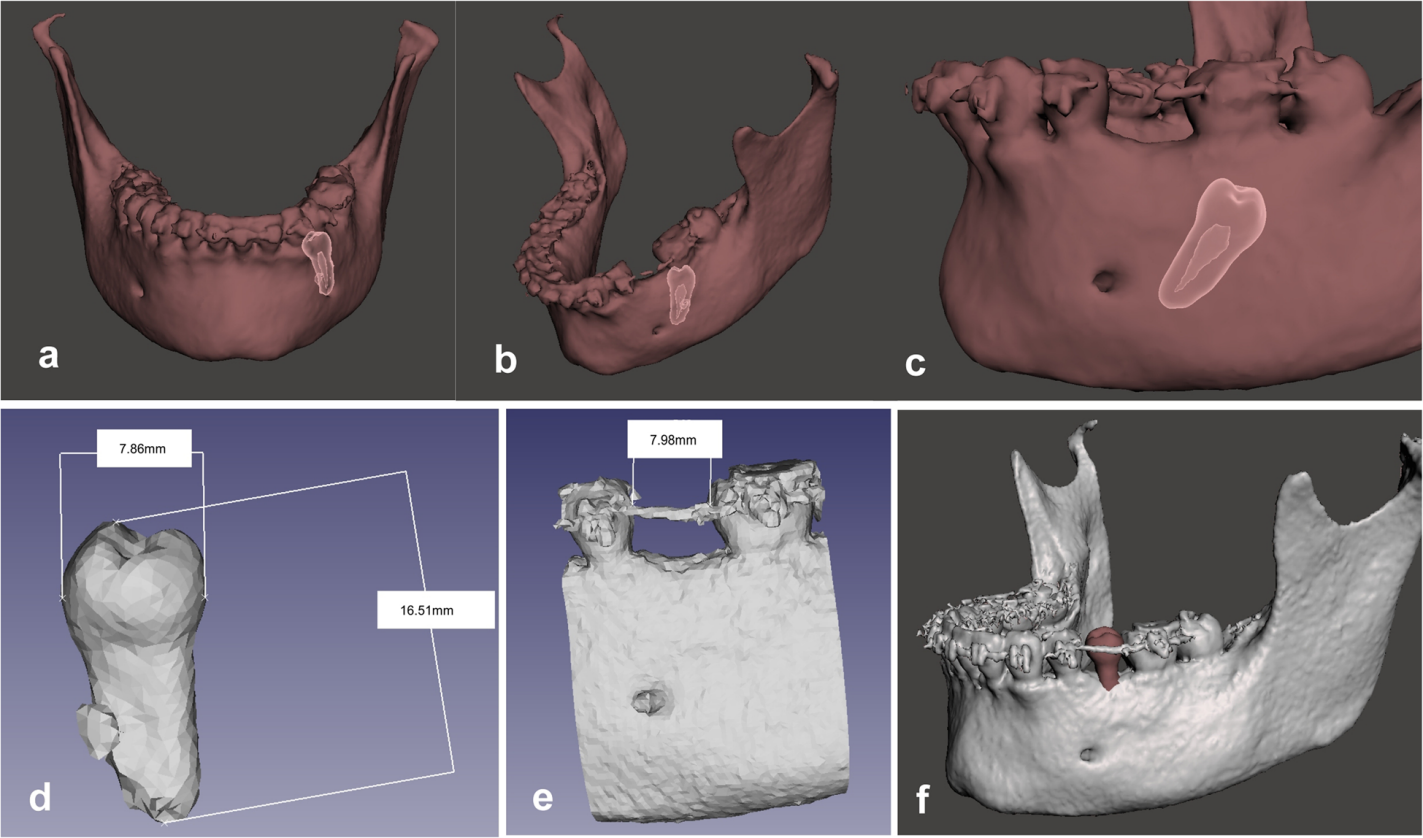
Virtual simulation of the autotransplantation of tooth 35 with Meshmixer free software (Autodesk®). **a–c** 3-D location of the ectopic tooth; **d–f** Measurements of the tooth to be transplanted, the space of the recipient site, and virtual positioning

To obtain the replica, helical CT images (Philips iCT256) with 58 mAs, kV 100 were used; radiation dose: DLP; 29.5 mGy-cm; CTDI vol (mGY): 4.9. After obtaining the image in DICOM format, it was segmented using the IntelliSpace Portal 11 program (Phillips®). This process allows the separation of a digital image into various structures, selecting the anatomical elements to be operated upon – in our case, the lower jaw and the ectopic tooth. Once the images were digitally segmented, in STL format, the surgery was planned using the Meshmixer program (Autodesk®), a 3D general design freeware that allows virtual planning of surgery on the basis of a post-processed model and the removal of the artifacts present in the CT caused by the brackets. It was also checked virtually means that the space available for the recipient socket was sufficient for the placement of the donor tooth (Fig. 2).

**Fig. 3:**
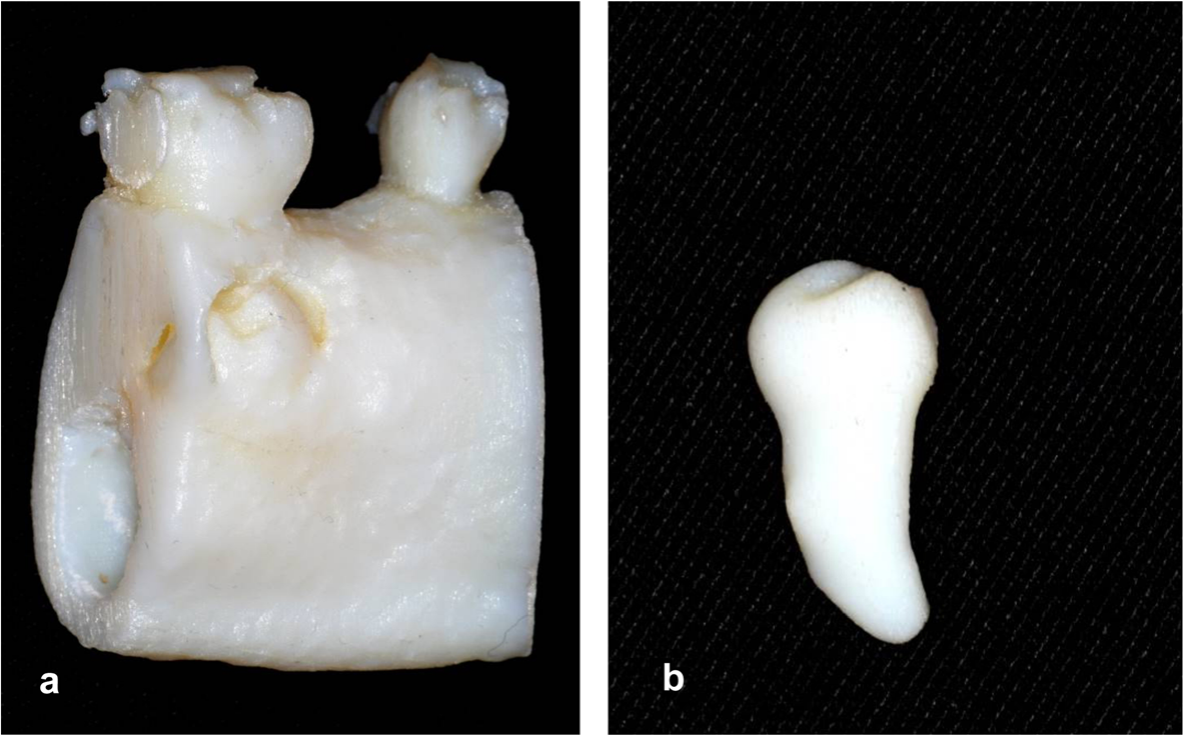
PLA and MED610 printing material and FDM and Polyjet technology. **a** Replica of the mandibular segment; **b** Replica of tooth 35

The Objet30 Prime® printer provided the dental replica and the Sigmar R19 (BCN 3D® Technologies) the mandibular segment, using PolyJet and FDM technologies respectively (Fig. 3). Med610 material (Stratasys®) was used for 3D printing of the dental replica and polylactic acid (PLA) for the printing of the mandibular segment and adjacent teeth. Before surgery, both the surgical replica of the ectopic tooth and the mandibular segment underwent low-temperature hydrogen peroxide plasma sterilization (< 50 °C; VH202 Matachana®).

**Fig. 4:**
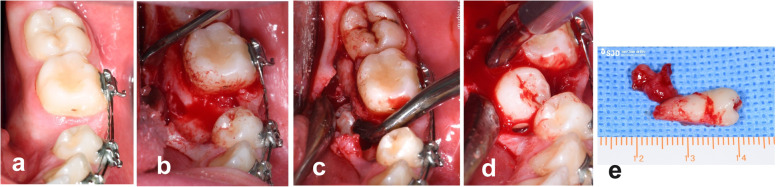
Oral surgery to extract tooth 35. **a** Pre-intervention; **b** Mucoperiosteal flap without lingual discharge; **c** fenestration of bone (lingual view); **d–e** Traction and avulsion of tooth 35

**Fig. 5:**
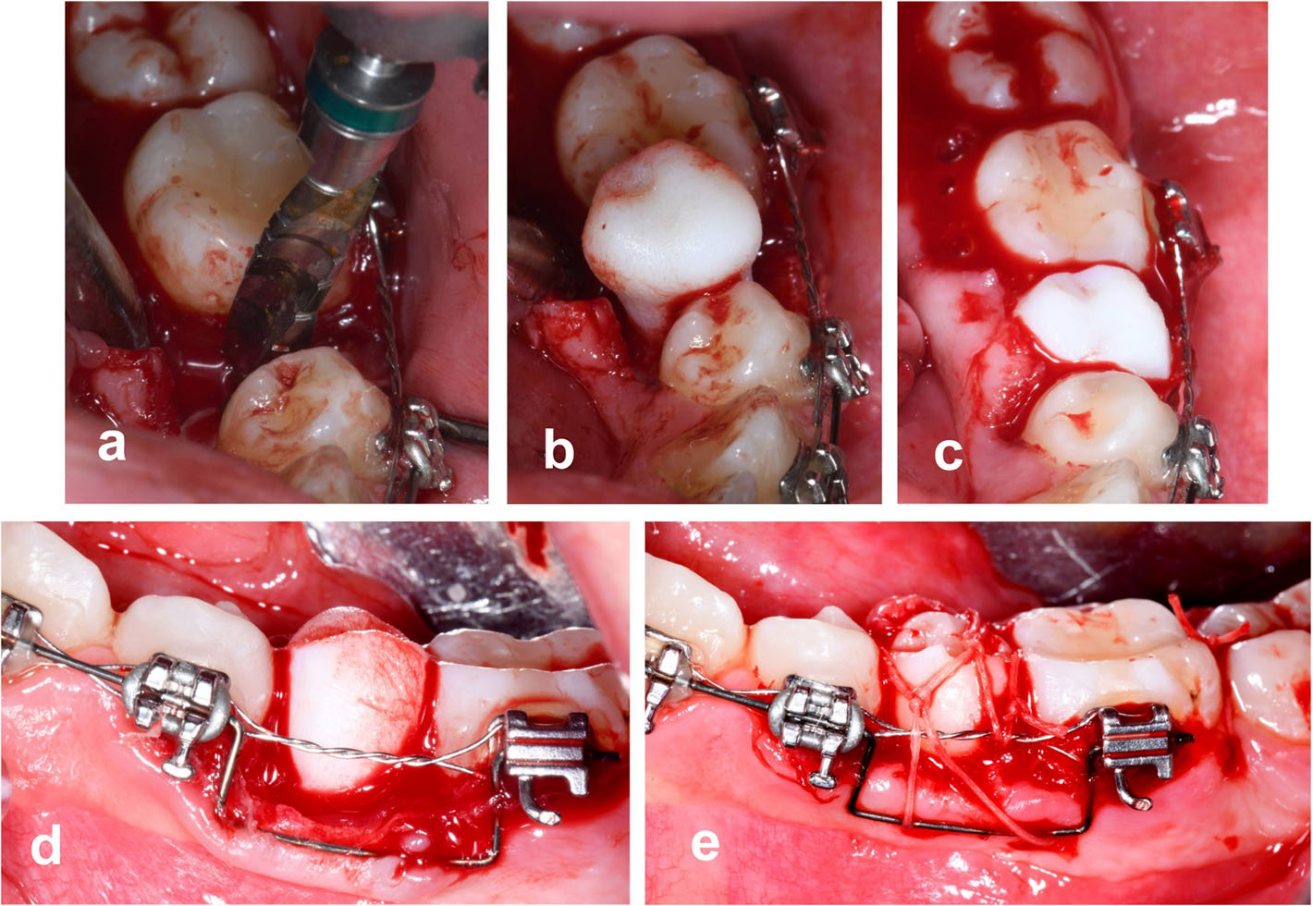
Autogenous tooth transplantation. **a** Grinding of the alveolar bed; **b** Testing of the surgical replica in the socket; **c** and **d** Placement of the replica in the socket (occlusal and sagittal view); **e** Placement of tooth 35 and stabilization by suturing

Given the difficulty of the surgery required to extract the donor tooth, it was decided to perform autogenous tooth transplant under deep sedation. A crestal and intrasulcular incision was made of adjacent teeth 34 and 36, without any discharge. Mucoperiosteal detachment of the lingual flap and bone fenestration were performed to allow careful extraction of tooth 35, which was immediately immersed in sterile saline solution (Fig. 4). Next, using a sequence of implant drills (MIS® Iberia), the recipient socket was created, 10 mm long and 5 mm in diameter. Throughout the process to check the position and stability, only the dental replica was used. We were obliged to perform minimal selective grinding of the replica tooth to correct a small mesiodistal discrepancy between teeth 34–36, which was also transferred to the donor tooth. After 15 min of extraoral time, tooth 35 was placed in its new receptor socket and stabilized with a Vicryl®4/0 cross suture (Fig. 5).

**Fig. 6:**
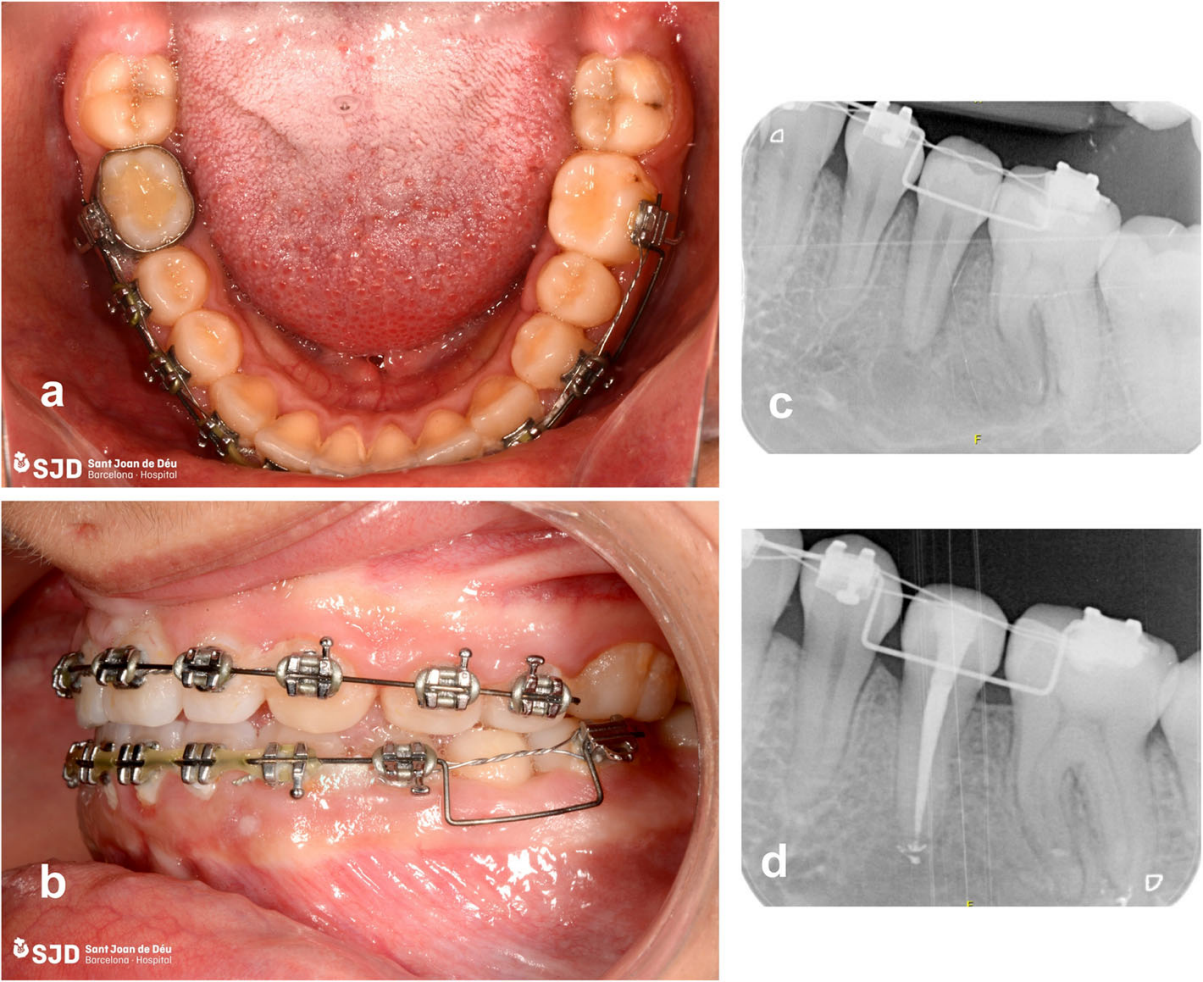
Clinical-radiological control. **a** and **b** Occlusal and sagittal clinical image; **c** and **d** Post-surgery periapical x-rays at 15 days and at 3 months with endodontic treatment complete

**Fig. 7:**
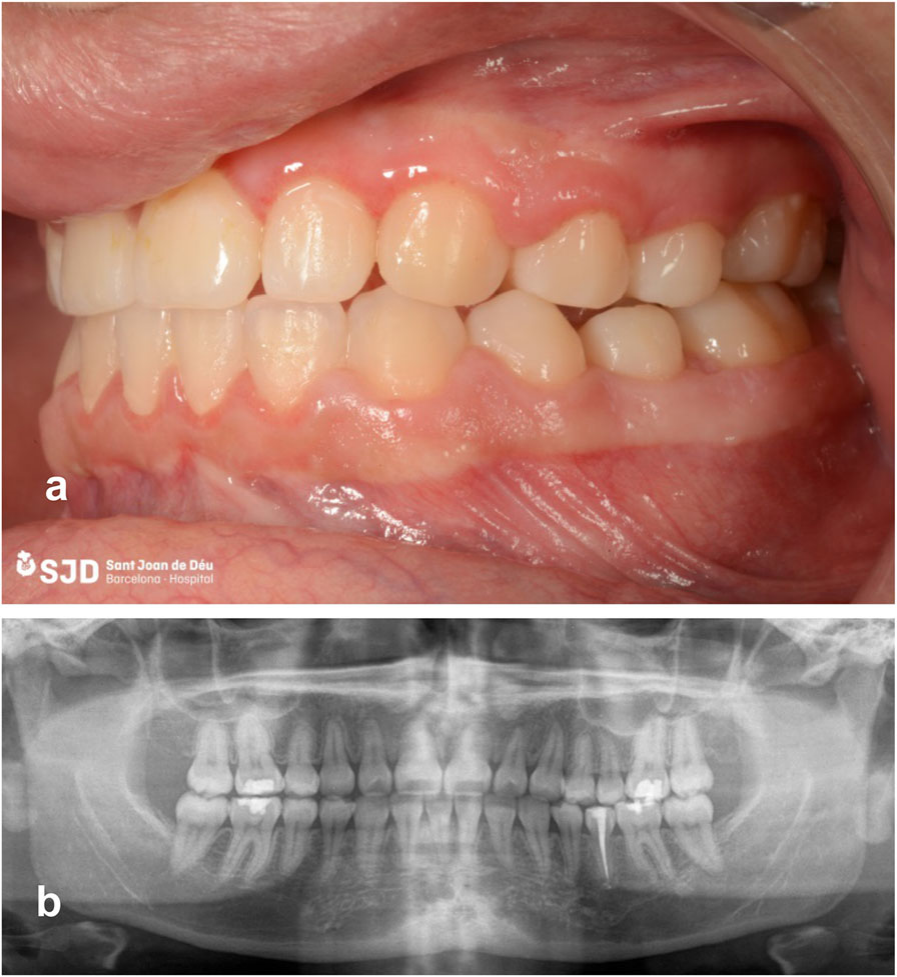
Clinical-radiological control. **a** Clinical control after the end of orthodontic treatment, at 12 months; **b** Final orthopantomography at 12 months

The patient received 7 days’ treatment with amoxicillin (500 mg every 8 h) and twice-daily chlorhexidine 0.12% rinses were indicated. Two weeks after the autotransplantation, endodontic treatment of premolar 35 was started (Fig. 6). Periodical clinical-radiological controls were performed at 1 month and at 3, 6 and 12 months (Fig. 7).

## Discussion

The use of 3D print technologies in medicine and dentistry is expanding rapidly. The range of applications is not limited to medical devices or prosthetics: as the development of bioprinting continues to evolve, the implantation of living tissues in regenerative medicine is now a possibility. 3D printing allows the production of a wide range of devices, from hearing aids to Invisaling® aligners to prosthetic limbs, all tailored to meet the specific needs of the patient. 3D printing has several applications in the different dental subspecialties. In oral and maxillofacial surgery, it offers a new approach to simulation and surgical planning. Prior to surgery, anatomical models provide a realistic impression. The concept “touch to understand” has caused a paradigm shift in the representation of anatomical models, which are now not just visual aids but visuo-tactile aids [14].

The treatment of ectopic tooth eruption varies according to the degree of severity: i) extraction of the deciduous tooth to facilitate the spontaneous eruption of the ectopic tooth, in cases with the depth of impaction < 5 mm and the inclination < 55º; ii) fenestration in combination with orthodontic traction when the depth of impaction is < 5.5 mm and inclination < 95º; and iii) autotransplantation, as the final therapeutic alternative to excision, if the depth of impaction is > 5.5 mm and without any inclination. Other aspects to consider are the patient’s age and ability to collaborate, the space available in the arch, and the presence of keratinized gingival tissue [16]. In our case, when analysing the impaction level of tooth 35, the distance was calculated using the 3D print of the mandibular segment. As it was above 6 mm, we decided to perform an autogenous dental transplant. In our case, the printing of the mandibular segment was necessary to design the shape of the recipient bed. Soon, using the mandibular replica, it would be interesting to prepare a surgical guide for these procedures. With this device resting on the adjacent teeth the surgeon is able to carry out guided surgery with better orientation and greater precision during the preparation of the new socket.

Kafourou et al. [17] reported the results of autogenous dental transplant according to two concepts, success and survival (Table 2).

**Table 2:** Tooth autotransplantation: success and survival criteria

Success	Survival
Teeth with immature root formation and pulp revascularization following transplantation	Asymptomatic replacement, resorption and unfavorable periodontal ligament healing.
Successful endodontic treatment performed (with absence of periapical pathogenesis): - teeth with necrotic immature root, - teeth with complete root.	Ankylosis: root surface merged with alveolar bone.
Favorable periodontal healing: - no evidence of external root resorption - resorption controlled with endodontic treatment. - normal alveolar bone process.	The tooth is still present in its transplanted position at the final follow-up visit.

A systematic review of the success of autotransplantation in teeth with incomplete root formation found that the donor tooth with the highest success and survival rates was the premolar (98.1%–98.4%), and that the most successful recipient area was the anterior area of the maxilla (98.5%) [18]. Other researchers analysing the same parameters, but in teeth with complete root formation, found the highest 5-year survival rates in the anterior teeth (96.9%), followed by the premolars (92.3%) and molars (84%) [19].

In our clinical case, the transplanted tooth was viable, with normal adjacent hard and soft tissues, and in stable occlusion; it was therefore considered successful. Other criteria assessed were the absence of progressive root resorption, discomfort, infection, physiological tooth mobility and a crown-root ratio ≤ 1.

The development of the 2 PM root is completed between the ages of 12 and 14. However, the ideal time for autotransplantation is when the apex of the tooth is still open with Moore’s stage 3–4, which favors its revascularization and complete root formation [19]. In our clinical case root development was already complete, and so endodontic treatment was required, as in the case of an avulsed tooth with a closed apex. Some authors recommend root canal treatment 14 days after tooth stabilization [19, 20]. Others, however, report a lower success rate of autotransplanted teeth with complete root formation [21].

In the literature, several researchers have reported factors that may influence the long-term results [22, 23]. Most agree that there are three variables that have a particularly negative influence on the survival of the donor tooth to be transplanted: i) damage to the periodontal ligament of the donor tooth by trying to fit it into the new alveolus in the recipient site; ii) excessive extra-alveolar time, which traumatizes viable periodontal ligament cells on the donor tooth; and iii) distance between the new alveolus and the root of the donor tooth [22–28].

According to Kafourou et al., the preservation of the periodontal ligament is the key factor in the prognosis of autotransplantation [17]. Whether or not an autotransplanted tooth requires splinting is a controversial issue. On the one hand, some authors consider that the mobility between the socket-tooth should be minimal in order to accelerate cell proliferation, reduce osteoclastic activity, and avoid possible occlusal trauma on the transplanted tooth [29]. Others, however, argue against splinting, in order to avoid pulp necrosis and inflammatory root resorption and to favor periodontal ligament repair [30, 31]. In our practice, we do not splint the transplanted tooth with the adjacent ones, but merely stabilize it with a surgical suture.

Several authors agree that teeth transplanted immediately or within 15–30 min of tooth extraction have the best prognosis [32, 33]. Lee et al. [11] were pioneers in applying additive manufacturing for the 3D printing of surgical dental replicas, in a study of 22 adult patients with clinical follow-up of 18 months. These authors highlighted the importance of reducing the extraoral time in donor teeth (mean 7.7 min) and of minimizing handling in order to preserve the fibers of the periodontal ligament [11]. In our case, the extraoral time was extended to 15 min because we had to correct a size discrepancy caused by the delay in performing surgery after the CT scan (a period of 4 months). In fact, the time between the CT and the surgery must be as short as possible, since slight orthodontic movements may cause small variations in the residual space. For all these reasons, orthodontic overcorrection is recommended to increase the space allocated to the recipient socket. Verweij et al. [34] recommend performing the cone beam-computed tomography scan between 2 months and 2 weeks before the autotransplantation procedure, because the time taken to create a 3D replica of the donor tooth is approximately 2 weeks.

Table 3 displays the 3D technique systems used in previous studies and the results obtained [11, 34–51]. Although resin is normally used as printing material for the replicas, some researchers have recently reported the use of titanium or cobalt-chrome alloys to make metallic replicas in order to prevent the development of deformities during the sterilization process [34, 48].

**Table 3:** Characteristics and outcomes of case reports and clinical studies

Study (year)	Study size (apex condition)	Results	AMT	Extraoral time (minutes)	Follow up time (months)
Lee et al. [11] 2001	22 undefined teeth	No sign of root resorption	SLA	3 to 17.5 Average: 7.7	18
Kim et al. [35] 2005	168 M 12 premolars & 2 others	81.9% completely healed. 9 were extracted	NR	Immediate to 25	2 to 60
Harzer et al. [36] 2009	1 premolar (o.a.)	Successful No signs of pathology	NR	NR	20
Honda et al. [37] 2010	1 lower third molar	Endodontic treatment. No signs of pathology	SLA	NR	48
Keightley et al. [38] 2010	1 premolar (o.a.)	Continued root formation No signs of pathology	SLA	< 1	6
Pang et al. [39] 2011	1 premolar (o.a.)	Successful No signs of pathology	NR	< 1	24
Lee & Kim [40] 2012	182 third molars (o.a and c.a.)	1.6% root resorption Other complications: NR	NR	Immediate to 25 Average: 7	NR
Park et al. [41] 2013	1 premolar (o. a.)	Successful No signs of pathology	NR	3	36
Jang et al. [42] 2013	5 M (o. a.)	Successful No signs of pathology	NR	Immediate to 2	24–90
Shabazian et al. [43] 2013	24 premolars	4 root resorption 4 ankylosis	SLA	< 1	12
Lee et al. [44] 2014	1 mesiodens (c. a.)	Successful No signs of pathology	NR	NR	36
Park et al. [45] 2014	2 M (c. a.)	Successful No signs of pathology	Polyjet	NR	10
Vandekar et al. [46] 2015	1 incisor (c.a.)	Endodontic treatment. No signs of pathology	NR	NR	12
Van der Meer et al. [47] 2016	1 premolar (o. a.)	Successful No signs of pathology	NR	Immediate	16
Verweij et al. [48] 2016	5 premolars (o. a.)	98% Successful No signs of pathology	SLM	less than 1 min	NR
Cousley et al. [49] 2017	1 premolar (o. a.)	Successful No signs of pathology	NR	less than 1 min	10
Kim et al. [50] 2019	2 third molars	Successful No signs of pathology	NR	3 and 5	2–60
Xia et al. [51] 2020	28 third molars	Successful No signs of pathology	SLA	1 to 5 Average: 2.5	24
Verweij et al. [34] 2020	73 premolars 24 M - 3 others	In 2016 98% Successful No signs of pathology	DMLS	82% less than 1 min	NR

*o. a.* open apex, *c. a.* closed apex, *NR* Non-Registered, *SLA* Stereolithography Apparatus, *Polyjet* Material Jetting, *SLM* Selective Laser Melting, *DMLS* Direct Metal Laser Sintering

The most frequent complications in autogenous tooth transplant are ankylosis, root resorption (inflammatory resorption) and pulp necrosis [18]. If present, ankylosis usually appears within 12 months of autogenous transplantation [31]; but some long-term follow-up studies indicated that this complication may also occur at a later time point [22, 52].

It is important to stress that the presence of pulp necrosis does not indicate surgical failure. In fact, endodontics is part of the autogenous tooth transplantation treatment plan [18]. For its part, progressive root resorption may appear due to injuries to the periodontal ligament and/or pulp tissue, which are usually seen on periapical x-rays between 2 months and 3 years after the autotransplant [32, 53].

## Conclusion

Autogenous tooth transplantation is a valid therapeutic alternative to extraction for resolving certain severe cases of ectopic tooth eruption. 3D additive manufacturing technology allows the preparation of a new recipient socket with the aid of a surgical replica of the tooth to be transplanted, thus minimizing handling and extraoral time.

## Data Availability

The datasets used and/or analysed during the current study are available from the corresponding author on request.

## Abbreviations

3D: Three-dimensional
2 PM: Mandibular second premolar
CT: Computed tomography
